# Case Report: An Intracranial Complication of COVID-19 Nasopharyngeal Swab

**DOI:** 10.5811/cpcem.2021.5.52232

**Published:** 2021-08-04

**Authors:** Alexandria Holmes, Bryant Allen

**Affiliations:** Carolinas Medical Center, Department of Emergency Medicine, Charlotte, North Carolina

**Keywords:** nasopharyngeal swab, meningitis, cerebrospinal fluid leak, meningocele, encephalocele

## Abstract

**Introduction:**

Cerebrospinal fluid (CSF) leaks are often the result of trauma or recent surgical procedures; however, a subset can develop from non-traumatic etiologies. Cerebrospinal fluid leaks from congenital and spontaneous encephaloceles can be clinically occult and have devastating consequences if undetected for prolonged periods of time. This report highlights a unique case of meningitis after CSF leak caused by ruptured congenital meningocele during a routine nasopharyngeal swab.

**Case Report:**

A 54-year-old female with diagnosed CSF leak presented to the emergency department (ED) with acute onset of severe headache, and neck and back pain. Prior to this presentation, the patient had experienced two months of persistent headache and rhinorrhea since her coronavirus disease 2019 (COVID-19) nasopharyngeal swab. As part of her outpatient workup, an otolaryngology consultation with subsequent beta-2 transferrin testing and magnetic resonance imaging was performed and she was diagnosed with a CSF leak from ruptured congenital meningocele. On ED presentation, she was afebrile, but with mild tachycardia, leukocytosis, and meningismus. Lumbar puncture revealed acute streptococcal meningitis. This patient’s meningitis developed due to prolonged occult CSF leak after her COVID-19 nasopharyngeal swab ruptured a pre-existing congenital meningocele.

**Conclusion:**

Nasopharyngeal swabs are being performed much more frequently due to the COVID-19 pandemic. All front-line providers should be aware of the potential presence and rupture of congenital meningoceles in patients who have undergone recent nasopharyngeal swab when risk-stratifying for potential CSF leak and meningitis.

## INTRODUCTION

Cerebrospinal fluid leaks (CSF-L) are rare but can occur in patients of all ages with potentially devastating consequences if not diagnosed and treated expeditiously. Most notably, the abnormal communication between the subarachnoid space and the extracranial space poses significant risk for meningitis. Meningitis, which often occurs within a year from onset of the leak, has been found to complicate almost 20% of patients with persistent leak with 10% mortality. 1 Most CSF-Ls encountered in the emergency department (ED) are in the setting of trauma or recent surgical procedures; however, it is important to recognize that leaks can also occur secondary to nontraumatic causes, including radiation, tumors, infections, or from either congenital or spontaneous encephaloceles. 2

Basal encephaloceles are lesions consisting of herniation of intracranial contents – meninges alone (meningoceles); brain tissue (meningoencephalocele); or a ventricle (hydroencephalomeningocele) – through a defect in the skull base. Congenital basal encephaloceles have an incidence of 1 in 35,000 live births (higher in Southeast Asia), but can also occur spontaneously. 3 Encephaloceles almost invariably present as a CSF-L; they are difficult to diagnose and often clinically occult until they result in potentially fatal complications such as meningitis, brain abscess, and sepsis. 2 Few cases of spontaneous or iatrogenic CSF-L and meningitis from encephaloceles have been described in the literature, and virtually none in the emergency medicine literature, especially pertaining to awareness of encephaloceles as a risk in development of CSF-L and meningitis. 3–9

We describe a case of a patient who presented to the ED with acute bacterial meningitis as a complication of CSF-L caused by ruptured meningocele during a routine nasopharyngeal (NP) swab for coronavirus disease 2019 (COVID-19).

## CASE REPORT

A 54-year-old obese female with a history of antiphospholipid syndrome on apixaban and recently diagnosed with CSF-L presented with acutely worsened headache and new-onset neck and back pain for one hour. She had photophobia, chills, and worsening of pain with any flexion of the spine. She denied a history of trauma, drug use, ill contacts, or recent travel. Two months prior, she had a NP swab of her left nare for COVID-19 during which she described a “popping sensation and intense pain.” Since the NP swab performance, the patient had experienced waxing and waning headache, left-sided rhinorrhea, post-nasal drip, cough, and intermittent fever. After failed therapies for pneumonia and allergies, she was referred to an otolaryngologist who performed a beta-2 transferrin test on her nasal discharge, confirming a diagnosis of CSF-L. After rigid nasal endoscopy was unrevealing for a source, magnetic resonance imaging (MRI) revealed small bilateral olfactory recess meningoceles (Image). She was scheduled for surgical repair and given appropriate return precautions that led her to present when she developed acutely worsened symptoms.

CPC-EM Capsule *Pending*

What do we already know about this clinical entity?

*Cerebrospinal fluid (CSF) leaks are relatively rare but do carry the complicating risk of secondary bacterial meningitis with a high associated mortality. Though rare, presence of encephalocele may increase risk of CSF leak as complication to nasopharyngeal (NP) swab performance*.

What makes this presentation of disease reportable?

*Incidence of basal encephaloceles is low at 1 in 35K live births. However, due to the widespread use of NP swabbing in the coronavirus disease 2019 pandemic, the potential exposure of these lesions to potential harm is great*.

What is the major learning point?

*Nasopharyngeal swab performance is not without risk, so care should be used educate performers on the appropriate technique. Onset of severe pain with persistent rhinorrhea after performance should prompt evaluation for CSF leak*.

How might this improve emergency medicine practice?

*Increased caution when performing NP swab may lead to decrease in complications, or earlier intervention for complications if recognized early*.

On ED presentation, the patient was afebrile, although mildly tachycardic. With the exception of meningismus, her exam, including neurologic exam, was normal. Her white blood cell count was 19 × 10^3^ cells/microliters (uL) (reference range: 4.1–10.7 × 10^3^ cells/uL) with a neutrophilic predominance. She was given intravenous (IV) dexamethasone, cefepime and vancomycin, and lumbar puncture was performed. The laboratory analysis revealed cloudy fluid, a glucose level of less than 10 milligrams per deciliter (mg/dL) (reference range: 40–70 mg/dL), protein level of 732 mg/dL (reference range: 15–45 mg/dL), and nucleated cell count of 2764/uL (reference range: 0–5 cells/mL) with 87% segmented neutrophils. The CSF Gram stain revealed Gram-positive cocci, and CSF culture grew *Streptococcus salivarius*. While awaiting admission, the patient developed a mild encephalopathy, which resolved over the subsequent 24 hours with continued antibiotic therapy.

During her hospitalization, the patient underwent bilateral endoscopic ethmoid repair with lumbar drain placement by otolaryngology and neurosurgery. Her hospital course was complicated by post-repair re-leak and development of ventriculitis. She had a ventriculostomy placed for treatment with a short course of intrathecal antibiotics in addition to a prolonged course of IV antibiotics. Once her CSF studies cleared, she underwent ventriculo-peritoneal shunt placement. Her clinical status improved, and she was ultimately discharged home with minimal neurologic sequelae.

## DISCUSSION

This patient had pre-existing, undiagnosed congenital meningoceles, the larger of which was iatrogenically ruptured during her NP swab for COVID-19, resulting in a CSF-L that remained undetected for two months until diagnosed by otolaryngology. While awaiting surgical repair, the patient ultimately developed meningitis requiring a lengthy hospital stay and prolonged antibiotic administration.

To our knowledge, this is the first reported case of a serious complication of a NP swab, despite performance instructions mentioning potential risk of injury. Nasopharyngeal swabs are routinely used for the collection of specimens from the surface of the respiratory mucosa to aid in diagnosis of infection, most often for influenza, respiratory syncytial virus, parainfluenza, and now COVID-19. There are no specific contraindications for performing a NP swab, other than exercising caution if the patient has undergone recent nasal trauma or surgery. 10 During the COVID-19 pandemic, as many as one million COVID-19 NP swabs per day were performed in the United States. 11 Relative to the one-in-35,000 incidence of congenital basal encephaloceles, plus additional spontaneous cases, providers must be aware of CSF-L as a potential consequence of such common testing, as the implications if undetected could be devastating.

This case also highlights congenital and spontaneous encephaloceles as potential risk factors for CSF-L and meningitis. Most emergency providers are aware of traumatic skull fracture and encephalocele, subsequent CSF-L, and risk for meningitis. This case argues that it is prudent for emergency providers to also consider the presence of often clinically occult congenital and spontaneous encephalocele(s) when risk-stratifying a patient for CSF-Land meningitis.

Congenital encephaloceles are due to a defect in the skull base usually resulting from defective ossification during development. Because of this, they are usually associated with other congenital anomalies. Providers should consider undiagnosed encephalocele in patients with a history of midline facial dysmorphisms, such as hypertelorism, broad nasal root, or cleft lip or palate, as well as optic disc anomalies. 3 Spontaneous encephaloceles are thought to occur primarily as a result of intracranial hypertension and exertion of hydrostatic pressure at anatomically weakened sites within the skull base, which act as release valves for the high pressure. 6 The presence of spontaneous encephaloceles should thus be considered in those patients at risk for increased intracranial hypertension, most commonly middle-aged, obese, hypertensive, multiparous women. 7

An additional takeaway from this case is the importance of recognizing the signs and symptoms of CSF-L to facilitate early diagnosis. The most common clinical symptoms of leak are headache, clear rhinorrhea or otorrhea, and salty post-nasal drip. 1 Patients are often mis-diagnosed with viral illness, the common cold, sinusitis, or allergic rhinitis. 5 Rhinorrhea and otorrhea in CSF-L often show positional dependency and are worse with valsalva maneuvers. Providers can look for the classic target or halo sign on gross assessment, which usually occurs when the CSF is mixed with blood or nasal discharge. A glucose oxidase test can also be performed on a sample of discharge; however, the test can often have false positive results when CSF is mixed with blood, and false negative results if meningitis has already developed. The most sensitive and specific test for detection is the beta-2-transferrin, which is only present in CSF, perilymph, and vitreous humor. 1 Only a 0.5-milliliter sample of discharge is required to detect the presence of beta-2-transferrin and confirm leak. 2

Once a leak is confirmed, the imaging modality of choice to evaluate for dural or osseous defect is most often high-resolution computed tomography (CT), which has a reported sensitivity of 89%. 1 That said, CT is less valuable in localizing cases of non-traumatic CSF-L, such as those associated with encephalocele, in which case magnetic resonance imaging may be more useful. 2 Cerebrospinal fluid leak can be managed conservatively with strict bed rest, head elevation to 30 degrees, and avoidance of blowing the nose, coughing, yawning, or straining; however, we know that longer duration of leakage and delayed repair have higher risk for meningitis. Early surgery is indicated in cases of penetrating injury, intracranial hematoma, meningitis, large intracranial aerocele, encephalocele, and low probability of success with natural repair. 1 There is controversy surrounding antibiotic prophylaxis while awaiting definitive management; however, to date there is little evidence to suggest that this provides any benefit. 12

## CONCLUSION

This case demonstrates the presence of an uncommon congenital anomaly that suffered injury during testing for COVID-19, complicated further by a secondary infection resulting in bacterial meningitis. Although such anomalies are rare, the drastic increase in the performance of nasopharyngeal swabs during the COVID-19 pandemic created an increased risk of iatrogenic injury and associated complication. Emergency physicians and other front-line providers should be aware of symptoms concerning for a cerebrospinal fluid leak following NP swab, as early identification and surgical intervention may help avoid serious complications.

## Figures and Tables

**Image:**
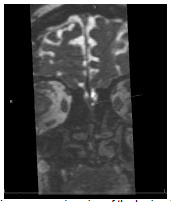
Magnetic resonance imaging of the brain without intravenous contrast, coronal slice. Small bilateral protrusions of cerebrospinal fluid below the level of the cribiform plate in the olfactory recess area of the nasal cavities bilaterally just ventral to the superior turbinates, left (arrow) slightly larger than right.
